# Development and validation of a distance-based training program of basic cardiac life support for laypersons and comparison with traditional in-person methodology: a non-inferiority study

**DOI:** 10.1186/s41077-026-00457-4

**Published:** 2026-07-03

**Authors:** Marcia Corvetto, David Acuña, Andrés Schneider, Jerónimo Rojas, Julián Varas, Elga Zamorano, Fernando Altermatt

**Affiliations:** 1 División de Anestesiología, Escuela de Medicina, Pontificia Universidad Católica de Chile, Marcoleta 367, Santiago, 8330024 Chile; 2 Departamento de Medicina de Urgencia, Escuela de Medicina, Pontificia Universidad Católica de Chile, Santiago, Chile; 3 Experimental Surgery and Simulation Center, Facultad de Medicina, Pontificia Universidad Católica de Chile, Santiago, Chile

**Keywords:** Simulation Training, Basic Cardiac Life Support, Cardiopulmonary Resuscitation, Distance-Based Simulation

## Abstract

**Background:**

This study aimed to determine whether a distance-based CPR training program with asynchronous feedback for laypersons is non-inferior to the traditional in-person Heartsaver® First Aid CPR AED course.

**Methods:**

After approval by the ethics committee, 192 non-medical personnel were recruited to participate in this study. Participants were randomly assigned to two different training methods:

- Traditional in-person Heartsaver® First Aid CPR AED course (T-course).
- Distance-based course offering asynchronous feedback through an online platform (D-course).

For the distance-based course, materials for practice were available at the participants' workplace (pad, resuscitation torso, an automated external defibrillator). The video-based assessment and feedback platform C1DO1 was used (https://c1do1.ai/). The course was structured in 9 stages on the platform, with theoretical and practical steps. Participants reviewed the videos and practiced unsupervised, uploading video recordings of their practice, and instructors assessed and provided feedback asynchronously on these videos. Participants then reviewed their own videos with the feedback and practiced again until approval.

Both groups completed a pre-training (PRE) and post-training assessment (POST). During both assessments, participants were recorded performing CPR. Videos were evaluated by two independent, blinded reviewers who rated participants’ performance using the AHA Heartsaver Adult CPR and AED skills testing checklist. Additionally, the quality of chest compressions (CC) was measured with the Prestan simulator application.

**Results:**

Of the 192 participants recruited, 172 completed the training, and 158 took the PRE and POST assessments. (83 finished the T-course and 75 the D-course).

Median Heartsaver Adult CPR and AED skills testing checklist scores increased from 2 (0–3) to 15 (14–16.5) points in the T-course and from 1 (0–2.5) to 16 (15.5–17) points in the D-course. The difference in POST assessment median scores between groups was -1 (95% CI: [-1.5, -0.5]), with the lower bound above the pre-established non-inferiority margin, confirming the non-inferiority of the D-course.

The median CC rate increased from 82 (0–106)/min to 105 (102–110)/min in the T-course and from 86 (0–109.5)/min to 105 (105–108)/min in the D-course. The median CC depth rose from 38 (0–57.5) mm to 58 (49.5–60) mm in the T-course and from 32 (0–56) mm to 59 (55–60) mm in the D-course.

**Conclusions:**

Both training programs significantly improve participants’ proficiency in CPR. The distance-based course with asynchronous platform feedback was non-inferior to the traditional Heartsaver® First Aid CPR AED course.

## Introduction

Cardiovascular diseases are the leading cause of death in Chile, accounting for 27% of deaths, according to the Department of Health Statistics and Information [1]. Particularly concerning is the increase in heart attacks among the young population, with a 34% rise in adults aged 30 to 50 years between 2017 and 2023, during which cases increased from 1,868 to 2,510 registered instances [1]. This trend underscores the growing need for quick and effective responses in cardiovascular emergency situations, especially when they lead to cardiopulmonary arrest (CPA).

Several reports, including those from the American Heart Association (AHA), indicate that patients’ prognosis following CPA depends directly on the activation of the emergency medical system, the early initiation of chest compressions (CC), and the quality of resuscitation [2–5]. Specifically, the survival rate after cardiac arrest decreases by 10% for every minute that passes without intervention, which highlights the importance of acting immediately [2]. The proportion of patients hospitalized with CPA and discharged alive increases significantly when bystanders initiate CPR within the first four minutes and deliver defibrillation within eight minutes [6].

In Chile, quick access to cardiopulmonary resuscitation (CPR) and automated external defibrillators (AEDs) is limited, especially in rural areas, where response times can be over 20–30 min [7]. Moreover, CPR training and AED use remain inadequate. A study in Santiago, Chile, showed that only 28% of patients with out-of-hospital cardiac arrest received bystander resuscitation [8]. Conversely, another study surveying physical education teachers about their resuscitation training found that 71% of respondents felt unqualified to perform CPR, despite 70% having previously attended training courses [9].

Since May 2019, Law 21.156 has been in effect in Chile, requiring high-traffic establishments to have AEDs. The law states that AEDs, due to their operational features and safety, allow individuals with minimal training to perform defibrillation. When combined with basic CPR maneuvers, this can save lives [10]. Although this law mandates certain public establishments to have defibrillators, implementing it faces major challenges [3]. The absence of widespread CPR training programs limits the impact of these devices, creating a significant gap between resource availability and their effective use during emergencies [11]. Unfortunately, reports have emerged of school staff failing to assist [12]. International experiences, like in Denmark, have demonstrated that mandatory CPR training programs for schoolchildren and drivers can substantially increase bystander resuscitation rates and improve survival outcomes in cardiac arrests [13, 14].

In this context, the study aimed to compare a distance-based CPR training program with asynchronous feedback for non-medical personnel to a traditional in-person Basic Life Support (BLS) course. The hypothesis was that the distance-based program would be non-inferior to the traditional course based on performance measures of CPR skills.

## Methods

### Study design

This was a prospective, randomized study aimed at comparing two different training methodologies: the traditional in-person Heartsaver® First Aid CPR AED course of the American Heart Association (T-course) and a distance-based course with asynchronous feedback through an online platform (D-course). The institutional review board approved this report and waived the need for informed consent, with approval number 221118007. (Comité de Ética en Investigación, Facultad de Medicina, Pontificia Universidad Católica de Chile). After approval by the ethics committee, 200 non-medical personnel were recruited to participate in this protocol. The study followed the CONSORT reporting guidelines.

### Participants and recruitment

The principal investigator contacted five different educational and sports institutions that had an AED defibrillator on their premises, explaining the project, and outlined the inclusion and exclusion criteria. Participants included in this study were adults (> 18 years old) with no health care background and capable to perform basic physical maneuvers, while the exclusion criteria included healthcare professionals and individuals who had previously completed CPR courses.

Each director of the five institutions provided the number of potential eligible participants from their organization. Participants received an invitation email explaining the protocol, and they could choose whether to participate. After assessing eligibility, a second email was sent describing the assigned group.

Participants were randomly assigned to two different training methods.

- Traditional in-person Heartsaver® First Aid CPR AED course (T-course).
- Distance-based course with asynchronous feedback via an online platform (D-course).

### Randomization

In this study, the institutions reported the number of participants who would be taking the course before the protocol began, so the entire list of participants was completely randomized at the beginning of the protocol.

Randomization was simple. The entire list of participants was completely randomized at the beginning of the protocol using the GraphPad Prism calculator. One of the researchers responsible for statistical analysis generated the random allocation sequence. This sequence was sent to the coordinator at the simulation center. We then emailed participants to inform them of their assigned group intervention and the next steps.

A total allocation concealment process was not possible in this study. Due to course implementation issues, participants knew which group they had been assigned to. The instructors who provided feedback in the distance learning course also knew. The evaluators of the pre- and post-course videos were blinded to the group assignments.

### Interventions

For the traditional in-person Heartsaver® First Aid CPR AED course offered by the American Heart Association, another simulation center with AHA certification was contacted to send the participants. Participants were scheduled for specific dates and times according to the agenda of the Simulation Center at Los Andes University in Chile to complete the certification. Offered by the AHA, the Heartsaver® First Aid CPR AED certification provides training in CPR, AED use, and first aid for individuals with little or no medical experience. Participants received an email from Los Andes University with the instructional materials and instructions for arriving on the day of their course. Those who did not attend their scheduled session were rescheduled a second time to reduce drop-offs.

**Fig. 1:**
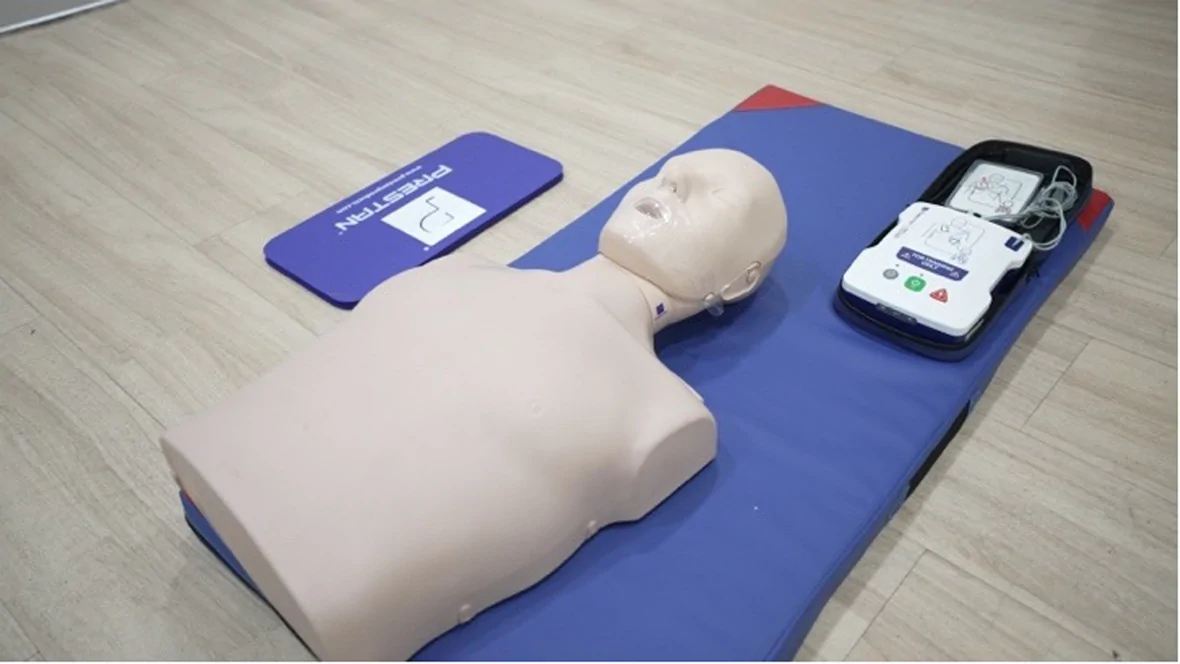
Materials for practice in the distance-based course (pad, resuscitation torso, and automated external defibrillator)

For the distance-based course, materials for practice—including a pad, resuscitation torso, and automated external defibrillator—were delivered by our simulation center staff to each institution, and a workstation was set up (Fig. 1). Depending on the number of participants from each institution, one or two workstations were established, allowing participants to practice at their workplaces during their free time.

To complete the course, the online platform C1DO1 was used (https://c1do1.ai/). This platform is a desktop and mobile application accessible from any location for participants and instructors with internet access [15]. Participants received an email to create a user account and gain access to the platform.

This distance-based course was designed by 6 experts (emergency medicine, anesthesiologist, nurses’ experts in education). These experts constructed and piloted this course during the COVID-19 pandemic using remote and asynchronous feedback [16]. The team tried to perform an equivalent course with the traditional in-person Heartsaver® First Aid CPR AED course regarding educational content, practical training time and cognitive load. The course was organized into stages on the platform, with both theoretical and practical components. It consisted of nine stages: five theoretical stages, three practical exercises, and a final stage with a multiple-choice assessment. The theoretical and instructional material stages were delivered through short video capsules. During practical stages, participants needed to practice and upload a video of their performance to the platform. It is required that participants complete each stage before moving on to the next. The course was designed to ensure that the objectives and training duration matched those of the BLS course. Each stage is briefly described in Table 1.

**Table 1:** Structure of the stages of the CPR course on the online platform

Stage 1	Introduction
Stage 2	Recognizing Cardiorespiratory Arrest
Stage 3	Quality of chest compressions
Stage 4	Practice stage: Quality chest compressions. At this stage, the student must upload a video to the platform
Stage 5	Use of Automated External Defibrillator (AED)
Stage 6	Practice stage: Use of AED: At this stage, the student must upload a video to the platform
Stage 7	Complete Resuscitation Sequence
Stage 8	Practice stage: Complete Resuscitation Sequence. At this stage, the student must upload a video to the platform
Stage 9	Multiple-Choice Test. The objective of this stage is to reinforce knowledge, it was not for research assessment purposes

**Fig. 2:**
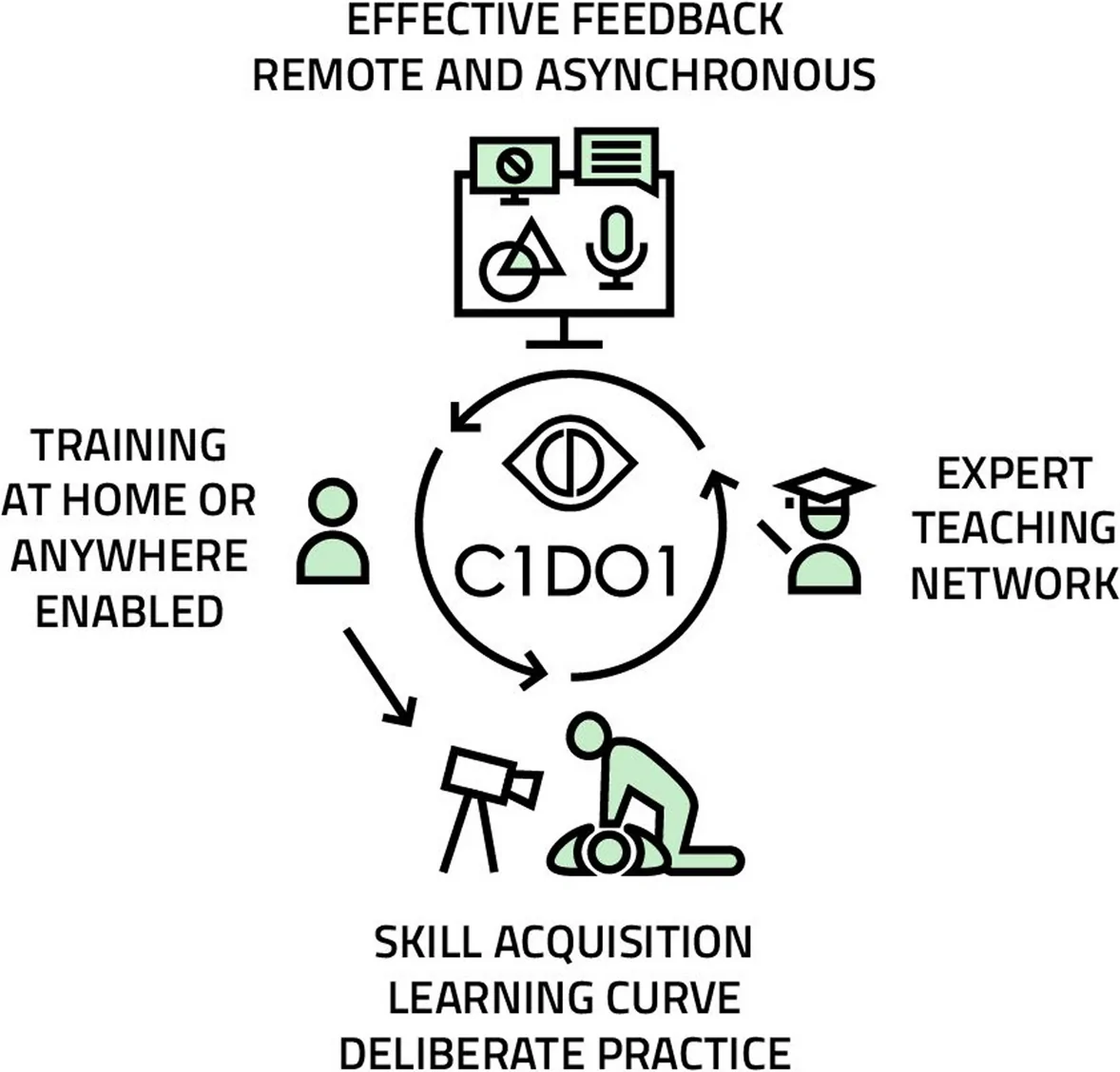
Asynchronous remote feedback method through the C1DO1 platform

The methodology used for distance-based courses has been applied in prior studies [15–18]. As mentioned above, the platform enabled students to access didactic materials on cardiopulmonary resuscitation and procedure-specific instructional videos for practice. Participants practiced and recorded themselves performing the procedure using a smartphone and uploaded their videos to the platform without supervision. In this study, participants had the practice station at their workplace. Instructors provided remote, asynchronous feedback within a 72-h window. Participants reviewed the feedback and practiced again until they received approval for the stage. The methodology for participant practice and instructor feedback through the platform is illustrated in Fig. 2. Feedback was provided by three instructors, all emergency medicine MDs, who are experts in healthcare simulation and feedback. The platform allowed them to give audio, written, and drawing feedback.

Finally, an online satisfaction survey was sent to participants to evaluate their perceptions after completing each training program.

### Outcome measures

The primary outcome was participant performance, assessed using the American Heart Association (AHA) HeartSaver Adult CPR and AED Skills Testing Checklist. The secondary outcome was chest compression quality, evaluated based on compression rate and depth.

Both groups completed a pre-training assessment (PRE) and a post-training assessment (POST). Our simulation center staff visited each institution to conduct the assessments for both groups. During each assessment, participants were videotaped by our staff during a CPR scenario. To ensure consistency, all participants watched a short video illustrating a situation at their workplace where a colleague becomes unconscious and needs medical attention. Later, two independent blinded reviewers evaluated the videos, rating the participants' performance using the AHA Heartsaver Adult CPR and AED skills testing checklist. The same assessment tool was used to evaluate both groups. These reviewers were different from the instructors providing feedback in the D-course. Additionally, the quality of CC was assessed using a Prestan® mannequin with a skill reporter connected to an application on a tablet device, which recorded the rate and depth of chest compressions.

### Definition of non-inferiority margins

Based on a pilot distance-based CPR course, the main outcome (Heartsaver Adult CPR and AED skills testing checklist) was expected to improve from 10–15% to 80–85% [16]. Previous studies testing clinical learning outcomes indicated that a 10–15% points non-inferiority limit is fair [19]. Concepts of non-inferiority in medical education have used examples like the comparison of Basic Life Support trainings and supported these limits [20]. Based on this data, it was decided to establish a difference of 2 points (performance difference of 10% between the trainings) as the non-inferiority margin for this study.

### Sample size calculation

A sample size of 156 subjects was calculated to detect a non-inferiority margin of 10% points on the checklist, with a significance level of 0.05 and a power of 0.8. The calculation was performed using the web calculator for non-inferiority trials provided by Sealed Envelope [21]. Considering this is a non-captive study population, meaning participants are not students of our institution, a 30% drop-off rate was considered. In a previous study with distance-based courses, the percentage of participants who finished and passed ranged from 20 to 60% depending on the course [15]. To ensure the adequacy of the calculated sample size, we ultimately assessed 200 participants for eligibility.

### Statistical analysis

The data were analyzed using JASP software version 0.19.3 for MacOS. Demographic data are presented as mean and standard deviation (SD). Checklist scores and chest compression (CC) data are shown as median and interquartile range (IQR). The intraclass correlation coefficient (ICC) was calculated to evaluate interobserver agreement.

Two-sided 95% confidence intervals (CI) were used to assess the significance of non-inferiority. Mann–Whitney U test and Wilcoxon signed-rank test were employed to compare independent and paired groups, respectively. A p-value of 0.05 was considered statistically significant.

Only participants who fully adhered to the study protocol, meaning they finished the assigned course and completed the POST assessment, were included in the analysis (Per-Protocol Analysis).

## Results

### Recruitment and baseline characteristics

After verifying the participants' eligibility with the participating institutions, a total of 192 non-medical personnel were recruited—96 for the traditional course and 96 for the distance-based course.

**Fig. 3:**
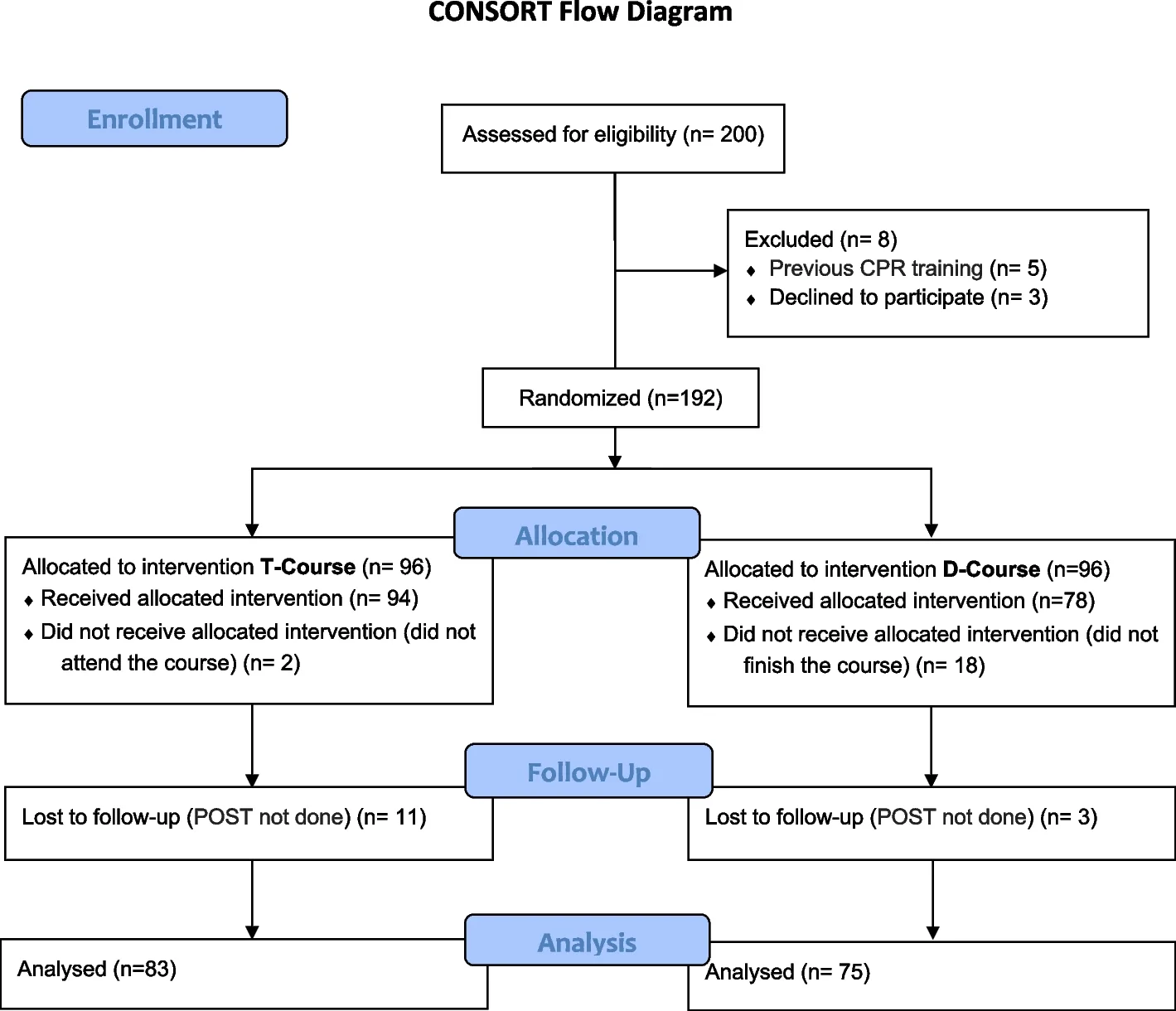
Consort flow diagram

Out of the total recruited, 172 participants completed the training: 94 finished the traditional course and 78 completed the distance-based course. Finally, 158 participants completed both the training and the PRE and POST assessments (Fig. 3).

Non-healthcare personnel such as teachers, psychologists, security guards, coaches, and athletes were recruited from five different educational and sports institutions. Demographic data from participants are presented in Table 2.

**Table 2:** Demographic data. Data are expressed in Mean ± SD or n (%)

	**T-course (*n* = 83)**	**D-course (*n* = 75)**
Age	43.1 (12.1)	37.7 (8.5)
Sex		
Male	39 (47)	34 (45.3)
Female	44 (53)	41 (54.7)
Institution from		
Educational institution	56 (67.5)	55 (73.3)
Sports institution	27 (32.5)	20 (26.7)
Profession or occupation		
Teacher	39 (47)	36 (48)
Psychologists	3 (3.61)	4 (5.33)
Security guards	5 (6.02)	4 (5.33)
Administrative	9 (10.84)	11 (14.67)
Coaches	6 (7.23)	4. (5.33)
Athletes	21 (25.3)	16 (21.33)

### Implementation of intervention

The traditional course was conducted at the Simulation Center of Los Andes University. The Heartsaver® First Aid CPR AED course offers 3 h of training by a certified instructor, including practice with a manikin using AHA materials. A total of 5 courses were held to accommodate all participants assigned to this group.

Regarding the distance-based course, participants accessed the platform and practiced at their workplaces as planned. From the moment they entered the platform, they were allowed to complete the course and exercises at their own pace for a period of 4 weeks.

### Primary outcome

**Fig. 4:**
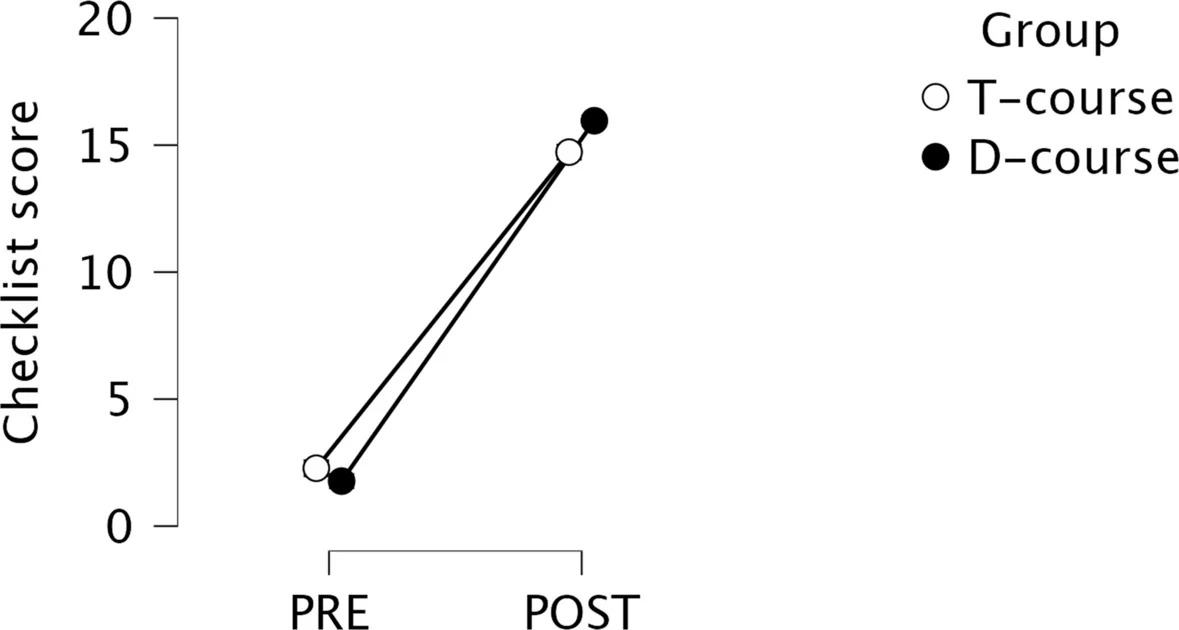
Median values of checklist scores in PRE and POST assessments of T-course (white) and D-course (black)

Median Heartsaver Adult CPR and AED skills testing checklist scores improved markedly from baseline in both groups (T course: from 2 [0–3] to 15 [14–16.5] points; D-course: from 1 [0–2.5] to 16 [15.5–17] points). The score improvements from the PRE to the POST assessment are shown in Fig. 4. Improvements were significant in both courses (*p* < 0.01) (Table 3). Regarding the difference between T-course and D-course, there was no difference in scores at the PRE assessment (*p* = 0.868) (Table 3).

**Table 3:** Median and IQR values of Checklist scores during PRE and POST assessments

	**T-course** (*n* = 83)	**D-course** (*n* = 75)	*P* value
Checklist PRE	2 (0–3)	1 (0–2.5)	0.868
Checklist POST	15 (14–16.5)	16 (15.5–17)	*p* < 0.01
*P* value	*p* < 0.01	*p* < 0.01	

**Fig. 5:**
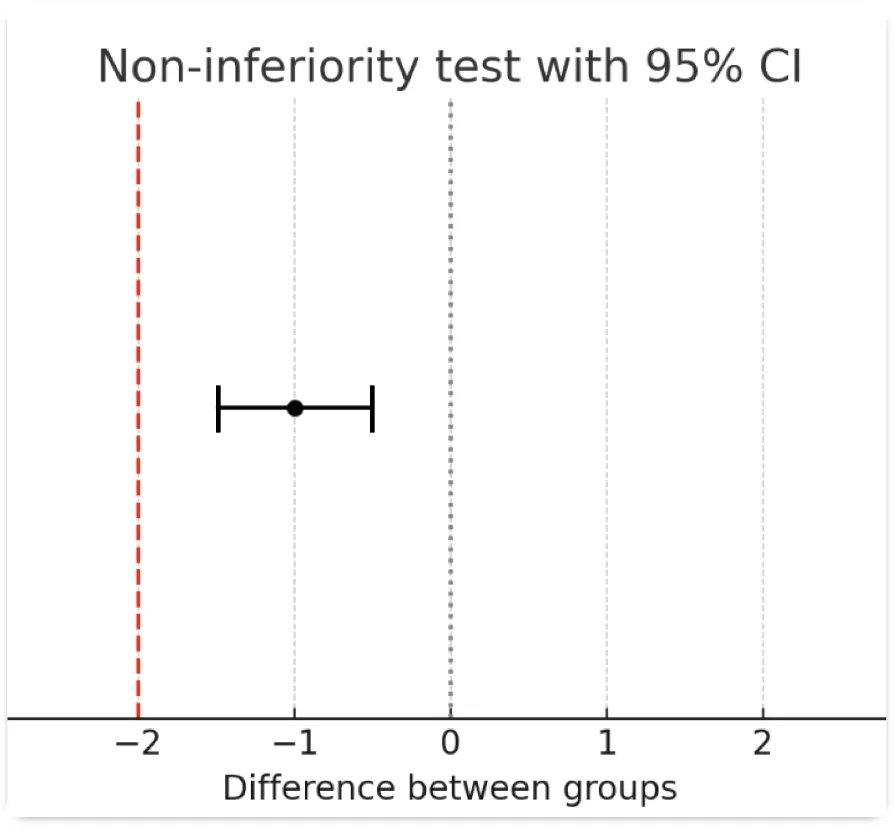
Non-inferiority results for overall performance measured with the AHA Heartsaver Adult CPR and AED skills testing checklist. Values < 0 favor T-course and values > 0 favor D-course. The black line indicates the lower and upper 95% CI of skills performance. The red line indicates the non-inferiority margin. Significant non-inferiority is given when the lower CI lies above this margin

The absolute difference in POST assessment median scores between groups was −1 (95% CI: [−1.5, −0.5]), with the lower bound above the predefined non-inferiority margin, confirming non-inferiority in the per-protocol analysis (Fig. 5). The absolute difference was calculated by subtracting the control group's POST assessment median scores from those of the new course.

Although the post-training median score was slightly higher in the distance-based group (*p* < 0.01), the effect size was small, and the difference stayed well within the non-inferiority threshold, showing that both training methods achieved similar levels of competence.

The interrater reliability for the observers' video Heartsaver Adult CPR and AED skills testing checklist scores was an ICC of 0.91.

### Secondary outcomes

The median CC rate improved from 82 (0–106) per minute to 105 (102–110) per minute in the T-course and from 86 (0–109.5) per minute to 105 (105–108) per minute in the D-course (Table 4). Both improvements were statistically significant (*p* < 0.01). Regarding the difference between T-course and D-course, there was no difference between groups in the PRE and POST assessments.

**Table 4:** Median and IQR values of Chest Compressions rate during PRE and POST assessments

	**T-course** (*n* = 83)	**D-course** (*n* = 75)	*P* value
CC rate PRE	82 (0–106)	86 (0–109.5)	0.640
CC rate POST	105 (102–110)	105 (105–108)	0.721
*P* value	*p* < 0.01	*p* < 0.01	

The median CC depth increases from 38 (0–57.5) mm to 58 (49.5–60) mm in the T-course and from 32 (0–56) mm to 59 (55–60) mm in the D-course (Table 5). Both improvements were statistically significant (*p *< 0.01).

**Table 5:** Median and IQR values of Chest Compressions depth during PRE and POST assessments

	**T-course** (*n* = 83)	**D-course** (*n* = 75)	*P* value
CC depth PRE	38 (0–57.5)	32 (0–56)	0.441
CC depth POST	58 (49.5–60)	59 (55–60)	0.044
*P* value	*p* < 0.01	*p* < 0.01	

Finally, the satisfaction survey shows high levels of satisfaction. Participants in both courses agreed or totally agreed with the statements asked (Table 6). Some interesting comments about the D-course concern the time interval for completing the course.

- *I would set a limit on the estimated training time.*
- *As a suggestion, I could offer a shorter timeframe. Since you left the timeframe open, it took me a long time to watch the videos.*
- *It would be better if you activate the course and evaluate it within a limited time frame.*

**Table 6:** Satisfaction Survey. Percentages of answers agree and totally agree in a 5-Likert scale

	**T-course (*n* = 83)**	**D-course (*n* = 75)**
Program's objectives of the course were clear	100%	96.5%
The instructional material provided promoted your learning	92.9%	93%
The evaluation conditions were communicated in a timely manner	98.2%	95.9%
Instructor’s feedback was fitting and centered on the learning objectives	98.2%	96%
Practical exercises seemed sufficient to me	96.4%	93.5%
The duration of the course was adequate	100%	100%
The platform used to develop the course easy to use	NA	90.7%
The instructional videos in the platform clearly explain the content	NA	96.5%
This course was important for me	92.9%	100%
Overall satisfaction with the course	100%	93%

## Discussion

This prospective randomized study aimed to demonstrate the non-inferiority of CPR training with a distance-based course that provides asynchronous feedback via the C1DO1 platform compared to the traditional BLS course.

The lower limit of the CI is greater than the non-inferiority threshold, so the results presented in this manuscript support the non-inferiority hypothesis within the established margin of 10% points of the checklist score (difference of 2 points). This means that the observed difference, even in its worst-case scenario (the lower limit of the CI), is no worse than the non-inferiority threshold. According to these findings, distance-based training with asynchronous feedback may be as effective as traditional face-to-face training for laypersons.

The acquisition of CPR competencies among non-medical personnel was very similar across both methodologies. These results are consistent with previous findings under similar training conditions. Skill improvement, measured by post-training CPR performance scores, is comparable to those reported in medical students [22] and other health science students [23]. In terms of chest compression quality achieved with the D-course, the parameters established as CPR quality metrics are met (CC rate of 100–120/min and CC depth of 50–60 mm) [24, 25]. Regarding training for laypersons, although assessment tools are diverse, skill improvement remains comparable [26, 27].

In education, several factors such as geographical barriers, costs, infrastructure deficiencies, and instructor availability may lead us to adopt a different or new didactic method for teaching a specific medical skill. That’s why a distance-based course can help extend CPR training to schools, universities, sports institutions, and other venues with large public attendance. Our main goal is to provide access to laypersons and help save the lives of patients experiencing cardiopulmonary arrest. In this context, before implementing the course broadly, we sought to determine whether this new method achieves a comparable level of learning success as the traditional standard approach [15].

Another argument supporting the search for distance-based training options is the greater organizational and financial burden typically associated with face-to-face training. In this study, some participants assigned to the traditional course (T-course) did not attend their scheduled sessions and had to be rescheduled to achieve the calculated sample size. Some participants required rescheduling up to three times, which ultimately increased the cost per student in this group.

Previous evidence about the global prevalence of basic life support training showed low rates of laypersons' training within the past two years, with significant regional variation in bystander training rates [28]. Socioeconomic status was a factor that correlates with the prevalence of bystander training. A systematic review and meta-analysis concluded that bystander training should be promoted worldwide [29]. Implementation of a targeted AED program in the Sao Paulo Metro subway system saved lives [30]. Supporting this evidence, some organizations, such as “The World Restart a Heart” (WRAH) initiative of the International Liaison Committee on Resuscitation, have pushed the message to save more lives with high-quality layperson CPR [31].

In response to this need, distance-based training could be a scalable method to reach remote and rural areas. It allows training programs to be easily accessible and available at any time or location. Additionally, this approach does not require the presence of expert trainers on-site. Despite these benefits and the growth of distance-based simulation over recent years, a recent review indicated that it remains in early development stages both methodologically and in terms of quality [32]. Specifically, some challenges were encountered in developing this type of course. Some participants started the course and progressed through several stages but did not complete it. Participants had the freedom to advance through the stages and perform practices within a 4-week period. During a satisfaction survey, three participants suggested that the course period could be shortened. Further research is needed to determine the optimal duration during which the course should be open to maximize completion rates. In this context, Ko et al. identified factors that can either facilitate or hinder laypersons' participation in resuscitation training [33]. Facilitators included prior experience witnessing someone collapse, awareness of automated external defibrillators in public places, certain occupations, and legal training requirements. Barriers to participation included personal factors such as advanced age, lower socioeconomic status, and educational background.

Another good example to highlight of the search for new methodologies to train laypersons in basic life support is the rise of virtual reality training. A recent systematic review showed that virtual reality and augmented reality-based training were as effective as traditional face-to-face CPR training [34]. Outcomes measured were similar to those presented in this study, such as chest compression rate, chest compression depth, and performance scores.

This study has several limitations. First, given the aim to train laypersons from educational and sports institutions, unfortunately participant characteristics were not controlled by researchers. Specifically, the study did not control variables that may substantially influence educational and practical performance outcomes, such us age, sex, educational level; digital or technological literacy, profession or physical ability. A limitation of this study is that it did not investigate the influence of participant heterogeneity on learning outcomes. Additionally, D-course used a remote and self-directed learning model, which may depend more heavily on digital literacy and learner autonomy.

Second, this project did not analyze the content of the feedback inputs in asynchronous feedback used (audio, written, or drawing) during the D-course. Despite the scheduled training sessions for the distance-based course, we do not know how much time the participants practiced or whether students continued training chest compressions after the second practical stage. Third, regarding chest compression quality outcomes, the Prestan® skill reporter used in this study did not assess parameters such as hand position and complete chest recoil. Given the nature and design of the study, only a per-protocol analysis was done. It is not possible to perform an intention-to-treat analysis, since the data of the post-course assessments are required. Finally, we did not perform a long-term retention assessment because it was not part of the research question.

## Conclusions

The distance-based course with asynchronous feedback via the C1DO1 platform was not inferior to the traditional Heartsaver® First Aid CPR AED course.

Since we have demonstrated the non-inferiority of this course, the next step for this project is to try to reach more users in sports and academic institutions throughout the country. Mass adoption of the course across the country will help us make progress toward this goal and improve bystander resuscitation opportunities in a country facing geographical and economic challenges.

Further research is needed to compare this distance-based approach with other emerging methods and ultimately evaluate the impact of distance-based training on real-world CPR performance and patient outcomes.

## Data Availability

The datasets used and/or analyzed during the current study are available from the corresponding author on reasonable request.

## Abbreviations

AHA: American Heart Association
CPA: Cardiopulmonary arrest
BLS: Basic Life Support
CPR: Cardiopulmonary Resuscitation:
AED: Automated external defibrillator
CC: Chest compressions
ICC: Intraclass correlation coefficient

## References

[1] Departamento de Estadísticas e Información de Salud (DEIS), Ministerio de Salud de Chile. Defunciones y mortalidad por causas, Chile 2022. Santiago: Ministerio de Salud de Chile; 2023. Available from: https://deis.minsal.cl/estadisticas-de-mortalidad/ Accessed 28 Apr 2026.

[2] Nazzal C, Lefian A, Alonso F. Incidencia de infarto agudo de miocardio en Chile, 2008-2016. Rev Med Chil. 2008;149(3):323–9. 10.4067/S0034-98872021000300323. 34479310

[3] Ministerio de Salud (MINSAL). Norma nacional de reanimación cardiopulmonar del adulto y pediátrica. Santiago: Ministerio de Salud de Chile; 2011.

[4] Chamberlain D, Smith A, Woollard M, Colquhoun M, Handley AJ, Leaves S, et al. Trials of teaching methods in basic life support (3): comparison of simulated CPR performance after first training and at 6 months, with a note on the value of re-training. Resuscitation. 2002;53(2):179–87. 10.1016/S0300-9572(02)00025-4. 12009222

[5] Sayre MR, Berg RA, Cave DM, Page RL, Potts J, White RD. Hands-only (compression-only) cardiopulmonary resuscitation: a call to action for bystander response to adults who experience out-of-hospital sudden cardiac arrest: a science advisory for the public from the American Heart Association Emergency Cardiovascular Care Committee. Circulation. 2008;117(16):2162–7. 10.1161/CIRCULATIONAHA.107.189380. 18378619

[6] Riva G, Ringh M, Jonsson M, Svensson L, Herlitz J, Claesson A, et al. Survival in out-of-hospital cardiac arrest after standard cardiopulmonary resuscitation or chest compressions only before arrival of emergency medical services. Circulation. 2019;139(23):2600–9. 10.1161/CIRCULATIONAHA.118.038179. 30929457

[7] Riggs M, Franklin R, Saylany L. Associations between cardiopulmonary resuscitation (CPR) knowledge, self-efficacy, training history and willingness to perform CPR and CPR psychomotor skills: a systematic review. Resuscitation. 2019;138:259–72. 10.1016/j.resuscitation.2019.03.019. 30928504

[8] Lara B, Chuecas J, Schild V, Musso J, Rojas J, Aguilera P. Registro prospectivo de pacientes que presentan paro cardiorrespiratorio extrahospitalario en Santiago, Chile. Rev Med Chil. 2022;150(10):1283–90. 10.4067/S0034-98872022001001283. 37358086

[9] Mayanz S, Barreto J, Grove X, Iglesias V, Breinbauer H. Paro cardiorrespiratorio extrahospitalario de causa cardiaca en Santiago de Chile: experiencia del equipo medicalizado del SAMU Metropolitano. Rev Chil Med Intensiva. 2009;24(1):9–16.

[10] Acevedo S, Córdova G, Clavería C, Larios G. Preparación de los colegios y profesores de educación física en prevención de muerte súbita y soporte vital básico. Rev Chil Cardiol. 2020;39(3):229–36. 10.4067/S0718-85602020000300229.

[11] Ministerio de Salud. Decreto 56: Aprueba reglamento sobre la obligación de disponer de desfibriladores externos automáticos portátiles en los establecimientos y recintos que indica, de acuerdo a lo establecido por la Ley No. 21.156. Santiago: Ministerio de Salud de Chile; 2020. Available from: https://www.bcn.cl/leychile/navegar?idNorma=1150508 Accessed 28 Apr 2026.

[12] T13. Niño está internado de gravedad tras recibir un pelotazo en el colegio: familia acusa falta de asistencia. T13 [Internet]. 2024 Aug 21. Available from: https://www.t13.cl/noticia/nacional/nino-esta-internado-gravedad-tras-recibir-pelotazo-colegio-acusan-falta-asisten-21-8-2024 Accessed 28 Apr 2026.

[13] Juul Grabmayr A, Andelius L, Bo Christensen N, Folke F, Bundgaard Ringgren K, Torp-Pedersen C, et al. Contemporary levels of cardiopulmonary resuscitation training in Denmark. Resusc Plus. 2022;1(11):100268. 10.1016/j.resplu.2022.100268. PMC9256815 35812720

[14] Malta Hansen C, Zinckernagel L, Ersbøll AK, Tjørnhøj-Thomsen T, Wissenberg M, Lippert FK, et al. Cardiopulmonary Resuscitation Training in Schools Following 8 Years of Mandating Legislation in Denmark: A Nationwide Survey. J Am Heart Assoc. 2017;6(3):e004128. 10.1161/JAHA.116.004128. PMC5523990 28292745

[15] Varas J, Belmar F, Fuentes J, Vela J, Contreras C, Letelier LM, et al. Improving medical student performance with unsupervised simulation and remote asynchronous feedback. J Surg Educ. 2024;81(12):103302. 10.1016/j.jsurg.2024.103302. 39442366

[16] Vera M, Kattan E, Cerda T, Niklitshek J, Montaña R, Varas J, et al. Implementation of Distance-Based Simulation Training Programs for Healthcare Professionals: Breaking Barriers During COVID-19 Pandemic. Simul Healthc. 2021;16(6):401–6. 10.1097/SIH.0000000000000550. 33913677

[17] Corvetto MA, Kattan E, Ramírez G, Besa P, Abbott E, Zamorano E, et al. Simulation-Based Training Program for Peripherally Inserted Central Catheter Placement: Randomized Comparative Study of in-Person Training With Synchronous Feedback Versus Distance Training With Asynchronous Feedback. Simul Healthc. 2024;19(6):373–8. 10.1097/SIH.0000000000000805. 38888993

[18] Corvetto MA, Kattan E, Varas J, Caro I, Altermatt FR. Designing Sustainable Solutions to Implement a Distance-Based Simulation Basic Life Support Training Program During COVID-19 Pandemic in Low-Income Countries. Simul Healthc. 2022;17(5):351–2. 10.1097/SIH.0000000000000651. PMC9560908 35260543

[19] Berg H, Steinsbekk A. Is individual practice in an immersive and interactive virtual reality application non-inferior to practicing with traditional equipment in learning systematic clinical observation? A randomized controlled trial. BMC Med Educ. 2020;20:123. 10.1186/s12909-020-02030-7. PMC7181571 32326948

[20] Klasen M, Sopka S. Demonstrating equivalence and non-inferiority of medical education concepts. Med Educ. 2021;55(4):455–61. 10.1111/medu.14420. 33206411

[21] Sealed Envelope Ltd. Power calculator for continuous outcome non-inferiority trial. 2012. Available from: https://www.sealedenvelope.com/power/continuous-noninferior/ Accessed 25 Feb 2020.

[22] Sianipar IR, Tantri AR, Muktiarti D, Dwijayanti A, Manggala SK, Muliyah E. Comparison Between Self-Deliberate Practice and Directed Learning Training Methods for Basic Life Support Knowledge and High-Quality Cardiopulmonary Resuscitation Skill Retention in Second-Year Medical Students 3 and 6 Months After Training. Med Sci Educ. 2023;33(2):395–400. 10.1007/s40670-023-01746-7. PMC10226953 37261012

[23] García-Suárez M, Méndez-Martínez C, Martínez-Isasi S, Gómez-Salgado J, Fernández-García D. Basic life support training methods for health science students: a systematic review. Int J Environ Res Public Health. 2019;16(5):768. 10.3390/ijerph16050768. PMC6427599 30832440

[24] Rao G, Savage DW, Erickson G, Kyryluk N, Lingras P, Mago V. Enhancing Cardiopulmonary Resuscitation Quality Using a Smartwatch: Neural Network Approach for Algorithm Development and Validation. JMIR Mhealth Uhealth. 2025;5(13):e57469. 10.2196/57469. PMC12089875 40324161

[25] Considine J, Gazmuri RJ, Perkins GD, Kudenchuk PJ, Olasveengen TM, Vaillancourt C, et al. Chest compression components (rate, depth, chest wall recoil and leaning): A scoping review. Resuscitation. 2020;1(146):188–202. 10.1016/j.resuscitation.2019.08.042. 31536776

[26] Bjørnshave K, Krogh LQa, Hansen SBa c, Nebsbjerg MAa, Thim T, Løfgren B. Teaching basic life support with an automated external defibrillator using the two-stage or the four-stage teaching technique. Eur J Emerg Med. 2018;25(1):18–24. 10.1097/MEJ.0000000000000410. 27203452

[27] González-Salvado V, Rodríguez-Ruiz E, Abelairas-Gómez C, Ruano-Raviña A, Peña-Gil C, González-Juanatey JR, et al. Training adult laypeople in basic life support. A systematic review Rev Esp Cardiol (Engl Ed). 2020;73(1):53–68. 10.1016/j.rec.2018.11.013. 30808611

[28] Gianotto-Oliveira R, Gonzalez MM, Vianna CB, Monteiro Alves M, Timerman S, Kalil Filho R, et al. Survival After Ventricular Fibrillation Cardiac Arrest in the Sao Paulo Metropolitan Subway System: First Successful Targeted Automated External Defibrillator (AED) Program in Latin America. J Am Heart Assoc. 2015;4(10):e002185. 10.1161/JAHA.115.002185. PMC4845117 26452987

[29] Ng TP, Eng SW, Ting JXR, Bok C, Tay GYH, Kong SYJ, et al. Global prevalence of basic life support training: a systematic review and meta-analysis. Resuscitation. 2023;186:109771. 10.1016/j.resuscitation.2023.109771. 36934835

[30] Elkin R, Duff JP, LaForest ML, Stapleton S, Ramachandra G, Palaganas JC, et al. Distance simulation in the health professions: a scoping review. Adv Simul (Lond). 2023;8(1):27. 10.1186/s41077-023-00266-z. PMC10656877 37978416

[31] Nakagawa NK, Lockey A, Carmona MJC, Hoover A, Nanda P, Böttiger BW. It is time to spread the message of high-quality layperson cardiopulmonary resuscitation all over the world. Clinics (Sao Paulo). 2024;79:100355. 10.1016/j.clinsp.2024.100355. PMC11066593 38678872

[32] Charnetski MD, Wawersik D, Palaganas JC, Duff JP, Bailey SKT, Ramachandra G, et al. Understanding the Effects of Health Care Distance Simulation: A Systematic Review. Simul Healthc. 2024;19(1S):S57–64. 10.1097/SIH.0000000000000760. 38240619

[33] Ko YC, Hsieh MJ, Schnaubelt S, Matsuyama T, Cheng A, Greif R. Disparities in layperson resuscitation education: A scoping review. Am J Emerg Med. 2023;72:137–46. 10.1016/j.ajem.2023.07.033. 37531710

[34] Sun R, Wang Y, Wu Q, Wang S, Liu X, Wang P, et al. Effectiveness of virtual and augmented reality for cardiopulmonary resuscitation training: a systematic review and meta-analysis. BMC Med Educ. 2024;24(1):730. 10.1186/s12909-024-05720-8. PMC11227211 38970090

